# Femoral Closure with Single ProGlide^®^ in Transcatheter Aortic Valve Implantation: A Registry-Based Study

**DOI:** 10.3390/jcm14197113

**Published:** 2025-10-09

**Authors:** Kévin Roulot, Marion Kibler, Antonin Trimaille, Adrien Carmona, Amandine Granier, Philoktimon Plastaras, Jérome Rischner, Stéphane Greciano, Pierre Leddet, Fabien De Poli, Mohamad Kanso, Ulun Crimizade, Karen Boyer, Minh Hoang, Michel Kindo, Laurence Jesel, Olivier Morel, Patrick Ohlmann

**Affiliations:** 1 Service de Cardiologie and Service de Chirurgie Cardio-Vasculaire, Pôle d’Activité Medico-Chirurgicale, Nouvel Hopital Civil, 67000 Strasbourg, Cedex, France; kevin.roulot@chru-strasbourg.fr (K.R.); marion.kibler@chru-strasbourg.fr (M.K.); antonin.trimaille@chru-strasbourg.fr (A.T.); adrien.carmona@chru-strasbourg.fr (A.C.); amandine.granier@chru-strasbourg.fr (A.G.); mohamad.kanso@chru-strasbourg.fr (M.K.); ulun.crimizade@chru-strasbourg.fr (U.C.); tam.hoangminh@chru-strasbourg.fr (M.H.); michel.kindo@chru-strasbourg.fr (M.K.); laurence.jesel@chru-strasbourg.fr (L.J.); olivier.morel@chru-strasbourg.fr (O.M.); 2 Hôpital Albert Schweitzer, 68000 Colmar, France; philoktimon.plastaras@diaconat-mulhouse.fr (P.P.); jerome.rischner@diaconat-mulhouse.fr (J.R.); 3 Hôpital Pasteur, 68024 Colmar, France; stephane.greciano@ch-colmar.fr; 4 Centre Hospitalier d’Haguenau, 67500 Haguenau, France; pierre.leddet@ch-haguenau.fr (P.L.); fabien.depoli@ch-haguenau.fr (F.D.P.); karen.boyer@chru-strasbour.fr (K.B.)

**Keywords:** transcatheter aortic valve implantation, registry, vascular access, femoral procedures, vascular closure device

## Abstract

**Background:** Vascular closure of the femoral artery during transcatheter aortic valve implantation (TAVI) remains a critical step prone to complications, despite advancements in introducer technology. The traditional technique involves using two ProGlide^®^ suture closure devices (2P), but alternative approaches, such as employing a single ProGlide^®^ device (1P), have emerged. **Aims:** We sought to evaluate the efficacy and safety of the 1P strategy compared to the standard 2P closure technique during transfemoral TAVI procedures. **Methods:** A registry-based study was conducted at the University Hospitals of Strasbourg, France, from January 2020 to December 2023. Consecutive patients who underwent TAVI via the transfemoral approach were deemed eligible. **Results:** The study cohort consisted of 1303 patients, with a mean age of 81.7 years and 47% female. The 1P strategy was used in 733 cases (56.3%), while the 2P technique was employed in 570 patients (43.7%). Hemostasis was achieved in the catheterization laboratory without additional devices in 30.4% of the single-ProGlide^®^ pre-closing cases. Vascular complication rates were similar in both groups, at 11.3% for the 1P technique and 11.4% for the 2P technique (*p* = 0.964). However, vascular closure device failure was significantly less frequent in the 1P group (1.6%) compared to the 2P group (5.3%). **Conclusions:** The 1P strategy for pre-closing during TAVI is as effective and safe as the conventional 2P approach. The 1P method offers potential advantages in terms of simplicity and cost-effectiveness.

## 1. Introduction

Aortic stenosis (AS) is the most common valvular disease in industrialized countries, with a significant epidemiological impact, particularly among the elderly, where its prevalence can reach up to 11% in individuals over 65 years of age [1]. Transcatheter aortic valve implantation (TAVI) has emerged as a transformative alternative to surgical aortic valve replacement [2] for patients with AS across various risk profiles, demonstrating comparable outcomes in terms of major clinical endpoints. Despite its efficacy, TAVI requires arterial access, typically achieved through a substantial arteriotomy. Notably, early procedures using first-generation valves [3] necessitated access sites up to 25 Fr, equivalent to approximately 8 mm in diameter [4]. Although advancements in valve design have reduced sheath sizes [5], the femoral approach remains the primary method for TAVI, necessitating effective closure techniques beyond manual compression [6,7]. In response, the medical community has developed and refined percutaneous closure strategies to address this challenge and minimize complications [8,9,10]. These efforts have led to significant improvements in vascular access management, driven by technological advancements and refined closure techniques [11,12,13,14,15]. Remarkably, innovations in introducer sheaths and closure devices have reduced vascular complication rates from approximately 20% in early TAVI experiences to less than 3% in recent studies [16,17,18,19].

Two main primary devices are utilized for closing large arterial access sites, including the suture-based Perclose ProGlide^®^ system (Abbott Vascular Devices, Santa Clara, CA, USA). The ProGlide^®^ system is typically employed using a dual-device technique (2P) at the procedure’s outset, as recommended for access sites larger than 8 Fr. However, since 2016, a single-device ProGlide^®^ pre-closure technique (1P) has been reported, offering potential benefits such as cost savings, reduced procedural time, and lower risk of vascular complications or stenosis associated with closure devices [16,20,21,22]. Several comparative studies have evaluated these techniques, though most involved smaller patient populations. At our institution, the 1P pre-closure technique was introduced in 2020 and has been increasingly adopted since its implementation. To evaluate its efficacy and safety in a large cohort of patients, we conducted this registry-based observational study. Specifically, we sought to compare this alternative strategy with the traditional 2P technique during transfemoral TAVI procedures, evaluating whether the 1P approach could achieve comparable or superior outcomes in terms of procedural success and complication rates, thereby potentially improving vascular access management.

## 2. Materials and Methods

### 2.1. Study Design and Reporting

We utilized data from a single-center registry of consecutive, unselected patients with AS who underwent transfemoral TAVI at the University Hospitals of Strasbourg, France, between January 2020 and December 2023. All patients provided informed consent prior to the procedure and agreed to the anonymous processing of their data as part of the FRANCE 2 and FRANCE TAVI registries. The registry (NCT01777828) was approved by the institutional review board of the French Ministry of Higher Education and Research and by the National Commission for Data Protection and Liberties (CNIL) with the reference DR-2012-640. This study was reported in accordance with the *Strengthening the Reporting of Observational Studies in Epidemiology* (STROBE) guidelines for observational studies [23].

### 2.2. Data Collection

Clinical data were collected through a thorough review of electronic medical records. The outcome of vascular closure was evaluated by the operator upon completion of the TAVI procedure. Vascular complications and hemorrhagic events were documented in two phases. During the procedure, they were reported directly by the medical team. Conversely, post-procedural complications were identified by the hospital coding department through a comprehensive analysis of electronic charts.

### 2.3. Procedure

Edwards Sapien-S3^®^ (Edwards Lifesciences, Irvine, CA, USA), Medtronic Evolut CoreValve^®^ (Medtronic Inc., Minneapolis, MN, USA), and Abbott Navitor^®^ devices (Abbott Vascular Inc., Redwood City, CA, USA) were implanted. The choice of device was at the discretion of the operator. Heparin was administered at the time of arterial puncture at a dose of 100 IU/kg, with additional boluses given to achieve an activated clotting time (ACT) of more than 250 s, which was maintained throughout the procedure. The decision to use a single-ProGlide^®^ (1P) or double-ProGlide^®^ (2P) closure strategy was left to the operator’s discretion and was not dependent on valve type, sheath size, vessel size, patient anatomy, or medical condition. In the traditional 2P strategy, two ProGlide^®^ devices were positioned at 10 o’clock and 2 o’clock orientations to ensure that the suture threads crossed at the center of the arteriotomy site, optimizing hemostasis through enhanced tissue approximation. In contrast, the 1P strategy employed a single device at 12 o’clock for direct closure. If the initial deployment of 1 or 2 ProGlide^®^ devices was insufficient to achieve hemostasis, additional device(s) were implanted with a 12 o’clock orientation. After successful hemostasis with the closure device(s), protamine was administered at a dose of 1 mg per 100 IU of heparin, followed by manual compression for 3–5 min.

### 2.4. Endpoints

We conducted an in-depth analysis of procedural reports to catalog the percutaneous closure devices used in each case and to detail the vascular and bleeding complications, which were classified according to the standardized Valve Academic Research Consortium-3 (VARC-3) criteria. The primary endpoint was a composite measure that included all vascular and bleeding complications—as comprehensively defined by the VARC-3 classification system—occurring during hospitalization. Secondary endpoints comprised (1) minor and major vascular complications, (2) bleeding events categorized according to the VARC-3 criteria, and (3) failure of the vascular closure system. Major vascular complications were defined as those resulting in death, VARC type ≥2 bleeding, limb or visceral ischemia, or irreversible neurologic impairment. These complications included aortic dissection or rupture, significant vascular injury, compartment syndrome, distal embolization (non-cerebral), unplanned endovascular or surgical intervention, or closure device failure. Minor vascular complications encompassed all vascular complications not meeting major criteria and not resulting in death, significant bleeding, ischemia, or neurologic impairment. Notably, the VARC-3 bleeding classification system includes four distinct types. Type 1 bleeding represents overt bleeding requiring medical intervention by healthcare professionals, hospitalization, or increased level of care but not requiring surgical or percutaneous intervention, equivalent to BARC 2 classification. Type 2 bleeding encompasses overt bleeding requiring one to four units of blood transfusion or associated with hemoglobin drop of 3–5 g/dL, corresponding to BARC 3a classification. Type 3 bleeding includes critical organ bleeding such as intracranial, intraspinal, intraocular, or pericardial bleeding with tamponade, bleeding causing hypovolemic shock requiring vasopressors, bleeding requiring reoperation or five or more units transfusion, or hemoglobin drop of 5 g/dL or greater, equivalent to BARC 3b, 3c, or 4 classifications. Type 4 bleeding represents fatal bleeding, either probable based on clinical suspicion or definite when confirmed by autopsy or imaging, corresponding to BARC 5a and 5b classifications, respectively. Failure of the vascular closure system was defined as the inability to achieve successful hemostasis at the access site, necessitating alternative interventions beyond planned balloon dilatation or manual compression, such as unplanned balloon use, stenting, or surgical intervention. Planned balloon dilatation at the arterial access site was employed prophylactically at the operator’s discretion for high-risk cases, including severe obesity, significant arterial calcification, traumatic arterial puncture, or other patient-specific factors that might compromise hemostasis. In contrast, unplanned balloon use constituted a rescue intervention following inadequate hemostasis with ProGlide^®^ device closure. Additionally, in-hospital mortality was evaluated as a secondary endpoint.

### 2.5. Statistical Analysis

Categorical variables are presented as frequencies and percentages, with comparisons performed using the chi-square test. Continuous variables are reported as means ± standard deviations and analyzed using one-way analysis of variance (ANOVA). All analyses were performed using SPSS, version 29.0.2 (IBM Corp., Armonk, NY, USA), with all tests two-sided at a 5% level of significance.

## 3. Results

### 3.1. Patient Characteristics

Table 1 presents the demographic and clinical characteristics of the 1303 study patients. The cohort had a mean age of 81.7 years, with a female prevalence of 47%. The distribution of valve types used in the study was as follows: Edwards^®^ Lifesciences LLC (Irvine, CA, USA) valves in 69.8% of cases, Medtronic^®^ (Minneapolis, MN, USA) in 26.1%, Abbott^®^ Vascular Devices in 3.1%, and Boston^®^ Scientific (Marlborough, MA, USA) in 1.1%. All femoral punctures were performed under ultrasound guidance to ensure precision, and general anesthesia was utilized in 1.9% of the TAVI procedures. The conversion rate to open surgery was minimal at 0.5%.

### 3.2. Study Outcomes

**Figure 1:**
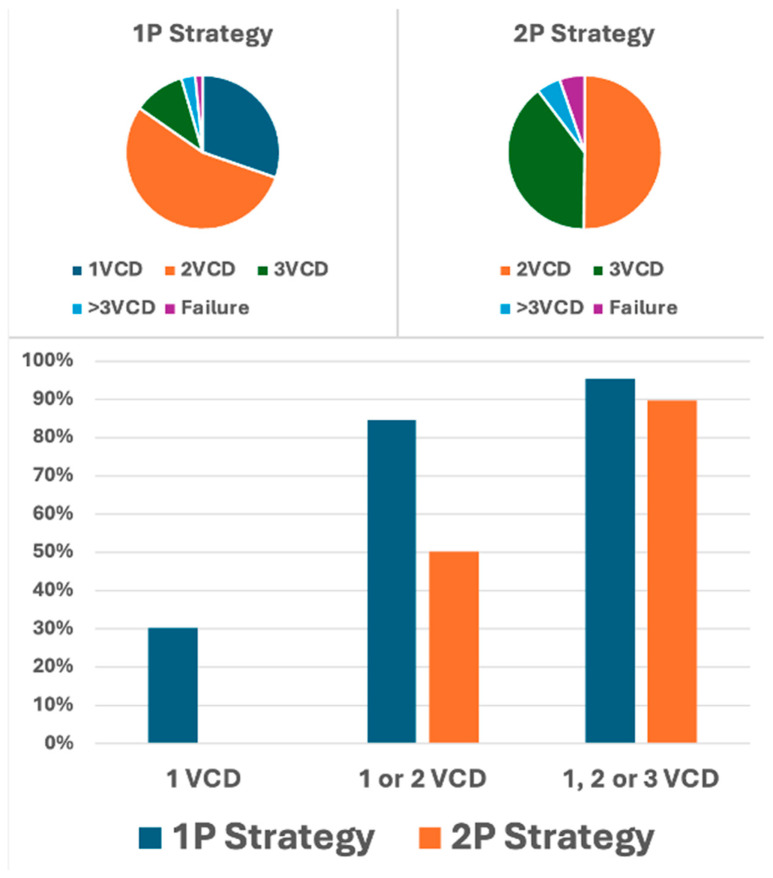
Distribution of vascular closure devices used per patient by pre-closing strategy for arterial access closure. **Upper panel**: distribution of vascular closure devices (VCD) used in the single-ProGlide^®^ (1P) and double-ProGlide^®^ (2P) strategies. The categories 1VCD, 2VCD, 3VCD, and >3VCD represent the percentage of patients requiring one, two, three, or more than three VCDs to achieve effective vascular closure, while “Failure” indicates the percentage of patients experiencing closure failure. **Lower panel:** percentage of patients achieving complete hemostasis with one VCD, one or two VCDs, or one, two, or three VCDs in both the 1P and 2P strategy groups.

**Figure 2:**
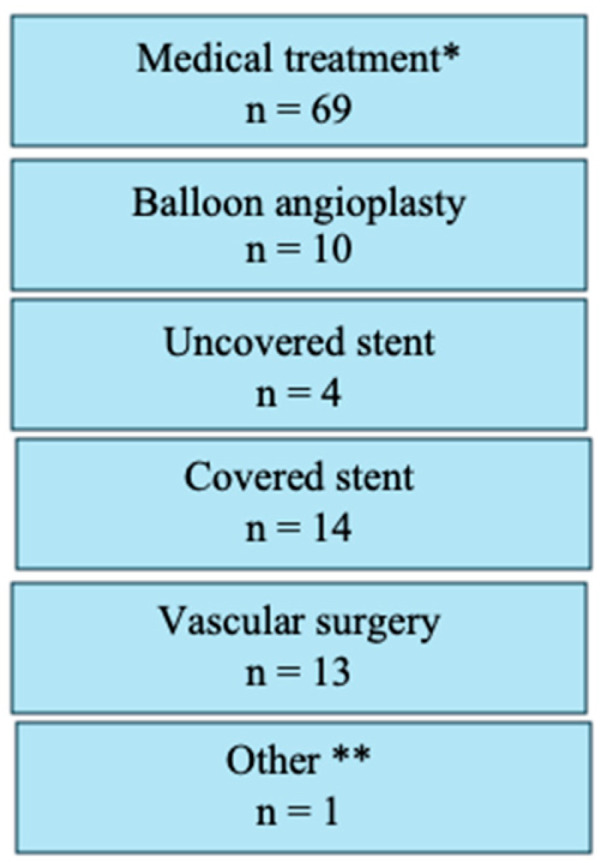
Treatment approaches for vascular and bleeding complications, showing the number of patients requiring various interventions including medical treatment, balloon angioplasty for hemostasis, uncovered stent implantation, covered stent implantation, vascular surgery, or other therapeutic approaches.

**Figure 3:**
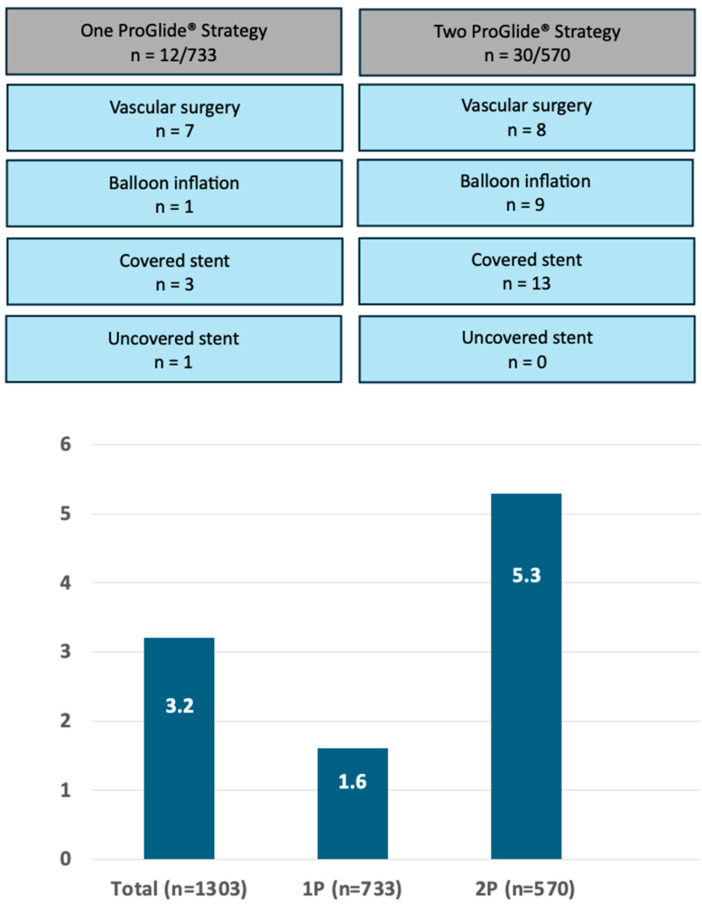
**Upper panel:** number of patients experiencing vascular closure failure in the single-ProGlide^®^ (1P) and double-ProGlide^®^ (2P) strategy groups, detailing complications treated by vascular surgery, balloon inflation, covered stent implantation, or uncovered stent placement. **Lower panel:** percentage of patients with device failure in the entire cohort and stratified by 1P and 2P strategy groups. The rate of failure of vascular closure devices was 3.2% among the 1303 patients treated via a percutaneous transfemoral approach for transcatheter aortic valve implantation. The failure rates were 1.6% with the single ProGlide^®^ strategy (1P) and 5.3% with the double ProGlide^®^ approach (2P) (*p* < 0.001).

**Figure 4:**
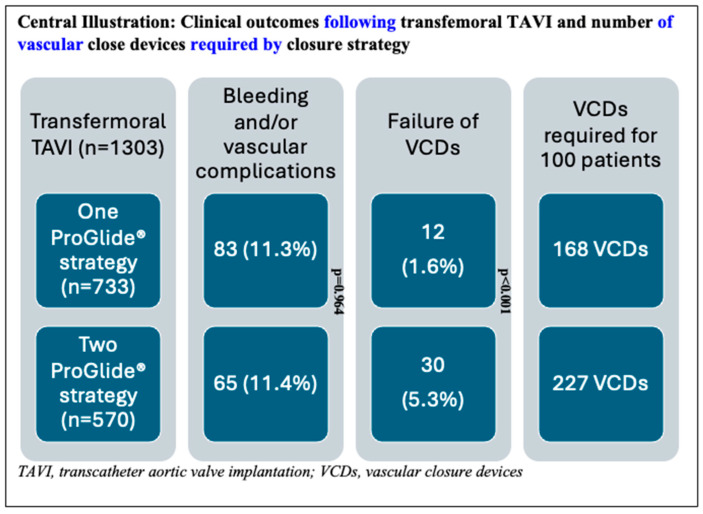
Description of the patient cohort showing the distribution of patients in the single-ProGlide^®^ and double-ProGlide^®^ strategy groups, the number and percentage of bleeding and vascular complications, vascular closure device (VCD) failure rates, and the number of vascular closure devices required per 100 patients treated.

For analysis, study participants were stratified into two distinct procedural groups: 733 patients (56.3%) underwent the 1P strategy, whereas 570 (43.7%) underwent the traditional 2P approach. In the 1P group, hemostasis in the catheterization laboratory was achieved without additional vascular closure devices (VCDs) in 30.3% of cases. However, a second VCD was necessary for 54.4% of patients, and a third VCD was required for 10.8% of the study participants. In contrast, the 2P strategy group required two VCDs for hemostasis in 50.2% of cases, whereas three VCDs were necessary for 39.5%. A comparison of the two strategies revealed that the 1P strategy involved the use of fewer devices overall compared to the 2P strategy (Figure 1). The primary endpoint, comprising bleeding and/or vascular complications during hospitalization, occurred in 11.3% (148/1303) of participants. Notably, no significant difference was observed between the two groups (1P, 11.3%; 2P, 11.4%; *p* = 0.964; Table 2). The occurrence of the primary endpoint showed no significant difference between women and men, both overall and when stratified by closure technique (1P versus 2P). The analysis of secondary endpoints revealed no significant intergroup differences in either minor (8.3% versus 8.1% in 1P and 2P, respectively; *p* = 0.870) or major (3.0% versus 3.3%, respectively; *p* = 0.733) vascular complications. However, minor type 1 VARC-3 bleedings were significantly more frequent in the 1P group (7.1%) compared to the 2P group (3.2%, *p* < 0.006; Table 2). There were no significant differences in the rates of type 2, 3, and 4 bleeding events among the groups. The management of the 148 (11.3%) vascular and/or bleeding occurrences during hospitalization included medical treatment (69 cases, 5.3%), balloon angioplasty (10 cases, 0.8%), uncovered stents for dissection (5 cases, 0.4%), covered stents for residual bleeding (33 cases, 2.5%), and surgical intervention (16 cases, 1.2%) (Figure 2). Closure device failure due to insufficient hemostasis or vascular complications occurred in 42 cases (3.2%), distributed as follows: 12 (1.6%) in the 1P group and 30 (5.3%) in the 2P group (*p* < 0.001; Figure 3 and Figure 4). The in-hospital mortality rate was 1.7%, with no significant difference among the groups (*p* = 0.085).

## 4. Discussion

In this single-center, registry-based study of patients undergoing TAVI for AS, the 1P strategy was found to be equally as safe as the 2P approach, with no significant difference in vascular and/or bleeding complications between the two groups. Notably, the 1P strategy achieved hemostasis using only a single VCD in 30.4% of cases. Furthermore, the number of VCDs required to attain hemostasis in the catheterization laboratory was lower with the 1P strategy compared to the 2P approach. Although minor type 1 VARC-3 bleeding complications were more frequent with the 1P strategy compared to the 2P approach, the failure rate of VCDs was significantly lower with the 1P technique.

To our knowledge, this is the largest report to date on patients undergoing TAVI with femoral access managed using the 1P approach. The incidence of major vascular complications in our large, unselected study population was consistent with those reported in the published literature. Specifically, our rate of 3.5% aligns closely with the findings of the PARTNER-3 trial (2%) and the Evolut Low Risk study (3.8%) [24,25]. In 2016, a pioneering comparison between the 1P and 2P strategies was conducted within the Japanese OCEAN-TAVI registry, involving a sample of 279 patients. The authors reported comparable vascular and bleeding complication rates for both groups, at 6.1% and 7.8%, respectively [20]. However, these rates were notably lower than those observed in our current report, where the complication rates were 11.3% and 11.4% for the 1P and 2P strategies, respectively. In 2022, Hollowed et al. [26] investigated a cohort of 489 patients, demonstrating lower vascular complication rates with the 1P strategy compared to the 2P approach (1.8% versus 4.9%, respectively). This research also showed a lower incidence of arterial dissections and stenosis exceeding 50% in the 1P group. In a separate study involving 1105 patients, Reifart et al. [21] reported comparable major vascular complication rates between the 1P and 2P strategies (9.2% versus 11.6%, respectively). Finally, the Cleveland registry, which included 740 patients, demonstrated comparable vascular complication rates between the two approaches [27]. Notably, the rate of successful procedures without the need for an additional device was 25% [27], a figure in substantial accordance with our findings (30.3%). In our study, type 1 minor VARC-3 bleeding complications were significantly more prevalent in the 1P group (7.1%) compared to the 2P strategy (3.2%, *p* < 0.001). Notably, VARC-3 bleeding complication type 1 includes overt bleeding requiring non-invasive treatment by healthcare professionals or transfusion of ≤ 1 unit of whole blood/red blood cells. In our institution, this category primarily represents access-site hematomas managed conservatively with compression therapy, which, in our clinical experience, have minimal impact on patient outcomes. The observed intergroup difference lacks a clear mechanistic explanation, and we believe this finding may represent a chance occurrence given the absence of other supporting procedural or patient-related factors. Of note is the fact that the MultiCLOSE study reported a remarkably low minor vascular complication rate of only 2.2% [16]. Notably, when considering the vascular access site and the success of vascular closure, the occurrence of VCD failure was significantly reduced with the 1P strategy, at 1.6%, compared to the 2P approach, which had a failure rate of 5.3% (*p* < 0.001).

In general, ultrasound guidance and operator experience are crucial in arterial closure procedures, as they play a significant role in reducing vascular complications [13,28,29]. In our study, ultrasound guidance was employed for all procedures, complemented by pre-TAVI CT scans, which are crucial for selecting the optimal access route to minimize the risk of closure complications. During these evaluations, factors such as anterior wall calcification, high bifurcation, tortuosity, and vessel diameter should be thoroughly assessed [30].

Our current findings advocate for the ProGlide^®^ single-device vascular closure strategy as a viable approach in unselected patients undergoing TAVI for AS. Notably, this technique is not only theoretically applicable to other percutaneous interventions involving large access points but also offers economic advantages. Specifically, the 1P approach markedly reduces the number of devices required, needing 168 devices for every 100 patients compared to 227 in the 2P strategy. This results in a saving of 59 devices and approximately EUR 9735, given the current cost of EUR 165 per ProGlide^®^ device in our institution. Furthermore, our data indicate that the sequential implantation of two ProGlide^®^ devices is more effective than simultaneous positioning, achieving success rates of 84% versus 50%, respectively. Several hypotheses may account for this difference. First, the ProGlide^®^ device may be more effectively applied when implanted singularly at the 12 o’clock position, perpendicular to the anterior wall of the artery. In contrast, placing two devices at the 10 o’clock and 2 o’clock positions may require manipulation or rotation, which can disrupt the optimal alignment of the device’s ‘feet’ and needles, potentially leading to suboptimal deployment. Additionally, in the 1P strategy, the second device is deployed at the 12 o’clock position over a puncture site whose edges have already been partially approximated by the first ProGlide^®^ unit. This may facilitate better wall application and edge approximation. To definitively validate the use of a single-ProGlide^®^ s trategy in the pre-closing phase, a randomized study is warranted, and ongoing trials (NCT06173115 and NCT05503199) are exploring this research question.

Our findings should be interpreted in the context of several limitations. This study is retrospective and observational in nature, making it inherently susceptible to confounding factors. The 1P and 2P approaches were carried out by different operators, potentially introducing selection bias. Furthermore, our dataset did not capture operator-specific information, preventing analysis of potential interactions between procedural strategy and individual operator performance. We also acknowledge that the temporal implementation of these strategies represents a potential confounding factor in our analysis. Historically, the 2P strategy was predominantly used in earlier years, while 1P implementation coincided with the initiation of our registry. Consequently, operators had extensive experience with the 2P approach but were on the learning curve for the 1P technique during early data collection. This experience differential may have contributed to higher complication rates in the 1P group, representing a limitation in the interpretation of our comparative outcomes. Moreover, the anatomical characteristics of the vascular access points, such as femoral artery diameter, calcifications, and tortuosity, may have had a significant impact on procedural outcomes. Similarly, the administration of anticoagulants during the procedure and protamine at its conclusion could have impacted the results. Nonetheless, their dosages were standardized across all groups. This uniformity implies that these variables were likely evenly distributed among the study groups, thereby reducing their potential to disproportionately affect the results. Moreover, our database did not capture detailed information on transfusion requirements. Specifically, transfusions of whole blood or red blood cells were not recorded separately. As a result, only predefined adverse events—including vascular complications (VARC major and minor) and bleeding complications (VARC types 1–4, adjudicated according to VARC-3 criteria)—were available.

## 5. Conclusions

This comprehensive study, comprising a large registry-based cohort of patients with AS undergoing TAVI, provides compelling evidence for the safety and efficacy of a vascular closure strategy utilizing a single ProGlide^®^ device. Our findings demonstrate comparable outcomes to the traditional two-device approach, with potential advantages in terms of cost-effectiveness and procedural simplicity. Whilst these results are highly encouraging, we emphasize the need for a large-scale, multicenter randomized controlled trial to definitively confirm these findings and establish this single-device strategy as a new standard of care in TAVI procedures.

## Figures and Tables

**Table 1:** General characteristics of the study participants (*n* = 1303).

Patient Characteristics	
Female sex, *n* (%)	613 (47)
Age, years [mean ± SD]	81.7 ± 7.5
Arterial hypertension, *n* (%)	693 (53.2)
Diabetes mellitus, *n* (%)	332 (25.5)
Dyslipidemia, *n* (%)	340 (26.1)
Active smoking, *n* (%)	43 (3.3)
History of coronary angioplasty, *n* (%)	262 (20.1)
History of coronary bypass surgery, *n* (%)	66 (5.1)
History of atrial fibrillation, *n* (%)	287 (22)
Chronic renal failure *, *n* (%)	158 (12.1)
STS score (%), [mean ± SD]	3.5 ± 3.7
**Procedural characteristics**	
Introducer sheath size	
14 Fr, *n* (%)	857 (65.8)
16 Fr, *n* (%)	267 (20.5)
18 Fr, *n* (%)	81 (6.2)
20 Fr, *n* (%)	87 (6.7)
21 Fr, *n* (%)	8 (0.6)
22 Fr, *n* (%)	3 (0.2)
Transcatheter aortic valve	
Self-expandable, *n* (%)	394 (30.2)
Boston^®^, *n* (%)	14 (1.1)
Medtronic^®^, *n* (%)	340 (26.1)
Abbott^®^, *n* (%)	40 (3.1)
Balloon expandable^®^, *n* (%)	909 (69.8)
Edwards Lifesciences^®^, *n* (%)	909 (69.8)
Secondary access	
Contralateral femoral artery, *n* (%)	606 (46.5)
Radial artery, *n* (%)	697 (53.5)

Data are presented as mean ± SD or counts (percentages), as appropriate. * Chronic renal failure was defined as an estimated glomerular filtration rate < 30 mL/min/1.73 m^2^. Abbreviations: SD, standard deviation; NYHA, New York Heart Association; STS, Society of Thoracic Surgeons.

**Table 2:** Clinical outcomes—defined according to the VARC-3 criteria—observed with the 1P and 2P strategies.

	Entire Cohort (*n* = 1303)	1P Strategy (*n* = 733)	2P Strategy (*n* = 570)	P
BC and/or VC	148 (11.3)	83 (11.3)	65 (11.4)	0.964
Major VC	41 (3.1)	22 (3.0)	19 (3.3)	0.733
Minor VC	107 (8.2)	61 (8.3)	46 (8.1)	0.870
Major VC at the primary access site for TAVI	24 (1.8)	13 (1.8)	11 (1.9)	0.83
Minor VC at the primary access site for TAVI	35 (2.9)	8 (1.1)	27 (4.7)	<0.001
Major VC at the secondary access site for TAVI	0 (0)	0 (0)	0 (0)	-
Minor VC at the secondary access site for TAVI	5 (0.4)	0 (0)	5 (0.9)	0.033
BC at discharge				
BC type 1	70 (5.3)	52 (7.1)	18 (3.2)	0.006
BC type 2	15 (1.1)	11 (1.5)	4 (0.7)	0.180
BC type 3	8 (0.6)	2 (0.3)	6 (1.1)	0.074
BC type 4	0 (0)	0 (0)	0 (0)	-
Death at discharge	23 (1.7)	17 (2.3)	6 (1.1)	0.085

Data are presented as counts and percentages. Abbreviations: VC, vascular complication; BC, bleeding complication.

## Data Availability

The original contributions presented in this study are included in the article. Further inquiries can be directed to the corresponding author.

## Abbreviations

The following abbreviations are used in this manuscript:

AS	Aortic Stenosis
TAVI	Transcatheter Aortic Valve Implantation
VCD	Vascular Closure Device
VARC-3	Valve Academic Research Consortium-3
ANOVA	Analysis of Variance
